# Supplementary material for: NXP031 Improves Cognitive Impairment in a Chronic Cerebral Hypoperfusion-Induced Vascular Dementia Rat Model through Nrf2 Signaling
Source: Int J Mol Sci. 2021 Jun 11;22(12):6285. doi: 10.3390/ijms22126285 (PMC8230952; doi:10.3390/ijms22126285)
Supplement: Supplementary file 1 [file ijms-22-06285-s001.zip › ijms-1243316-supplementary.pdf]

Contents

Figure S1. Pre-behavior test in radial 8-arm maze test p. S2

Figure S2. Full gel pictures from Fig 3 p. S3

Figure S3. Full gel pictures from Fig 4 p. S4

Supplementary Figure S1. Pre-behavior test in the radial 8-arm maze test

8 wks post-BCCAO

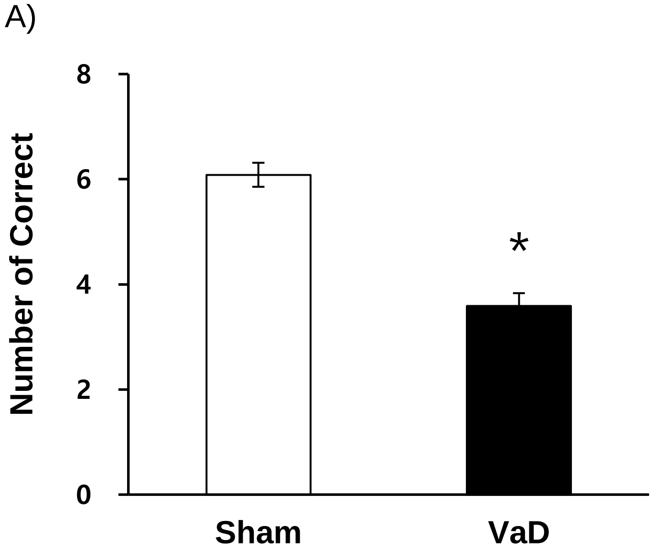

Figure S1. Cognitive impairment induced by BCCAO was confirmed using a radial 8-arm maze test. A) The VaD group show significantly decreased the number of correct choices compared to the Sham group. B) In addition, the VaD group was increased the time spent on each arm of the maze. The data are presented as the mean ± S.E.M. *p<0.05, compared with the Sham group.

Supplementary Figure S2. Full gel pictures from Fig 3

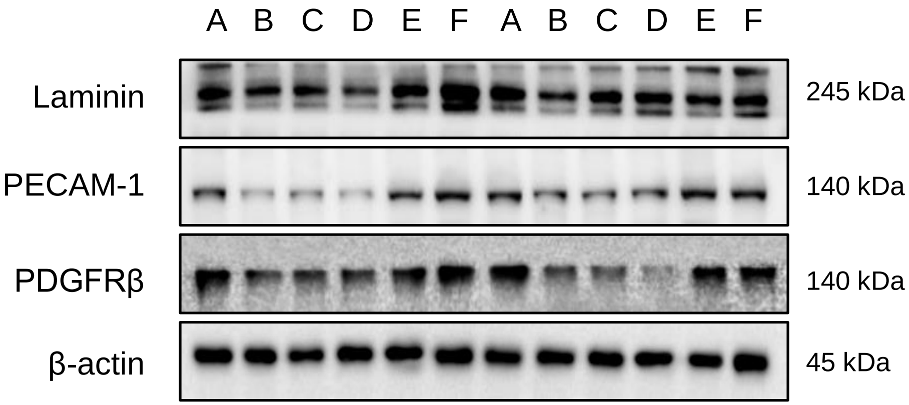

Figure S2. Representative bands of Laminin, PECAM-1, PDGFRβ protein expression in the hippocampus after CCH. (A) Sham, (B) VaD, (C) VaD + Vitamin C, (D) VaD + Aptamin, (E) VaD + NXP031_M, (F) VaD + NXP031_H.

Supplementary Figure S3. Full gel pictures from Fig 4

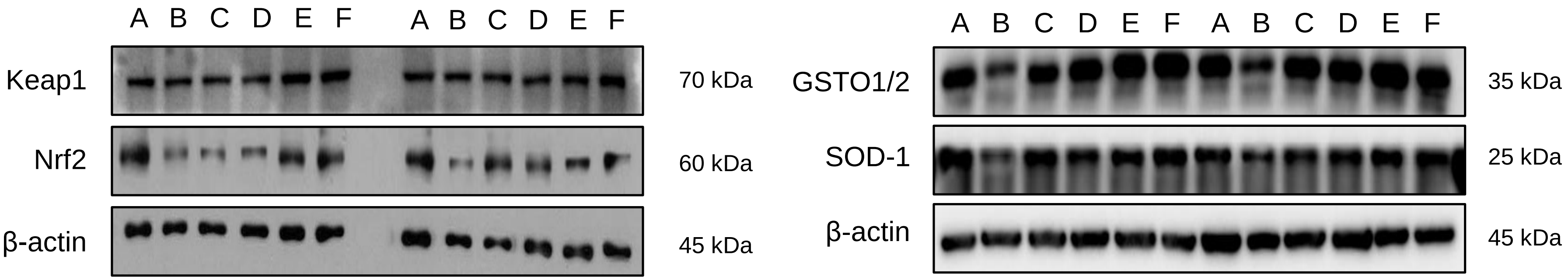

Figure S3. Representative bands of keap1, Nrf2, GSTO1/2, and SOD-1 protein expression in the hippocampus after CCH. (A) Sham, (B) VaD, (C) VaD + Vitamin C, (D) VaD + Aptamin, (E) VaD + NXP031_M, (F) VaD + NXP031_H.
